# Chronicles of Cell Death Foretold: Specificities in the Mechanism of Disposal

**DOI:** 10.3389/fimmu.2017.01743

**Published:** 2017-12-11

**Authors:** Lindsey D. Hughes, Lidia Bosurgi, Sourav Ghosh, Carla V. Rothlin

**Affiliations:** ^1^Department of Immunobiology, School of Medicine, Yale University, New Haven, CT, United States; ^2^I. Medizinische Klinik und Poliklinik, Universitätsklinikum Hamburg-Eppendorf, Hamburg, Germany; ^3^Bernard-Nocht-Institut für Tropenmedizin, Hamburg, Germany; ^4^Department of Pharmacology, School of Medicine, Yale University, New Haven, CT, United States; ^5^Department of Neurology, School of Medicine, Yale University, New Haven, CT, United States

**Keywords:** apoptosis, homeostasis, tissue repair, phagocytic receptors, signal integration

## Abstract

Massive turnover of cells occurs through apoptosis during the constant remodeling of our tissues at homeostasis, from the shedding of cells at exposed barrier surfaces to the elimination of autoreactive lymphocytes. However, a surge of apoptotic cells also accompanies tissue damage, infection, and inflammation. A salient feature of apoptosis in either scenario is the exposure of phosphatidylserine (PtdSer) on the outer leaflet of the plasma membrane. In response to this cue, a range of phagocytes are charged with the sizeable task of engulfing apoptotic bodies and disposing of the billions of cells that perish each day. The presence of apoptotic cells in the remarkably distinct immunological settings described above, therefore, raises the question of how phagocytes are able to coordinate appropriate responses to apoptotic cells—from their silent removal to the production of growth factors or tissue repair molecules—following such a ubiquitous signal as PtdSer exposure. Here, we consider several emergent properties of phagocytes and apoptotic cell clearance that may facilitate specification among this suite of potential responses.

## Introduction

“…but in this world nothing can be said to be certain, except death and taxes.” (Benjamin Franklin)

Apoptotic cell death is a perennial component of tissues from embryonic development through to adulthood. Is death the great equalizer in which all apoptotic cells are handled identically by phagocytes? Or is every instance of apoptosis unique—the trigger and context of a particular cell death event granting specificity to the phagocyte response? During many instances of developmental apoptosis, such as digit formation or postnatal pruning of neuronal circuits, there is simply a need to cull. Conversely, in the case of apoptosis following tissue damage or injury, there is a fundamental need to replace the dying cells; disposal must be linked to regenerative signals. Viral infection of cells can also trigger death by apoptosis, such that the indiscriminate uptake of this cell could threaten the phagocyte with infection. Does knowledge of why cells are undergoing apoptosis enable us to foretell the nature of their removal? Are there distinct mechanisms of identification in effect to enable this functional diversity? In this review, we discuss the view that the array of phagocytes, their repertoire of receptors, the temporal expression pattern of these receptors, and coincidence detection mechanisms may confer specificity to the disposal of dead cells.

## Phagocyte and Receptor Diversity: Redundant or Requisite?

### Molecular Basis of Apoptotic Cell Recognition

Phagocytes rely on a set of specific, conserved morphological changes in apoptotic cells in order to recognize that these cells are dying and to initiate their selective and swift clearance. These permissive “eat-me” signals include, but are not limited to, oxidized low-density lipoproteins, surface-bound thrombospondin, ICAM-3, calreticulin, C1q, and phosphatidylserine (PtdSer) exposed on the outer leaflet of the plasma membrane (1). Most “eat-me” signals are byproducts of the activation of intracellular cysteine-dependent aspartate-directed proteases (caspases), found downstream of both the intrinsic and extrinsic initiators of apoptosis [molecular pathways reviewed in Ref. (2, 3)]. Additional modifications to these surface alterations, such as the oxidation of PtdSer lipids containing linoleic or arachidonic acids, may serve to minimize the aberrant removal of cells that transiently expose PtdSer while activated (4, 5).

Phagocytes express numerous receptors that identify each apoptotic “eat-me” signal and instigate the rearrangement of the cytoskeleton to engulf the apoptotic corpse. In mammals, a large cohort of receptors carry out apoptotic cell sensing, with upwards of 10 cell surface receptors capable of recognizing exposed PtdSer either directly or indirectly through bridging molecules (1). The importance of apoptotic cell clearance in preventing systemic autoimmunity may provide sufficient biological impetus for maintaining redundant receptors. Nevertheless, as discussed below, phagocytic receptors that appear interchangeable based on their activating signal may in fact harbor differences in their downstream signaling components, expression profiles, regulation, and interactions with other receptors that provide the basis for diverse phagocyte responses following exposure to apoptotic cells.

It should be noted that recent advances have also distinguished additional forms of programmed cell death, including necroptosis and pyroptosis, which engage distinct signaling pathways, but yet also parallel the lytic dissolution seen in necrosis (6, 7). Loss of cell membrane integrity in each of these cases results in the release of molecules typically restricted in the nucleus or cytosol, which can then be sensed as damage-associated molecular patterns by phagocytes. For example, SAP-130, a component of the U2 small ribonucleoprotein complex, and F-actin unleashed from necrotic cells have each been established as ligands for innate immune receptors (8–11). The identification of signals that are uniquely released or expressed by cells that die through these distinct modalities of necrosis remains an area of active research.

### Dynamics of Phagocytic Receptor Expression

The expression profile of phagocytic receptors varies notably among phagocyte populations, and may be further modulated by the surrounding microenvironment. The developing embryo represents one setting of persistent apoptotic cell death and efferocytosis without concomitant inflammatory signals. Extensive cellular proliferation and apoptosis is required for proper limb remodeling, organogenesis, and formation of synapses throughout the nervous system. Mice lacking even single components of the apoptotic signaling pathways exhibit severe developmental defects, often leading to perinatal lethality. For instance, Hao *et al*. utilized a knock-in approach to express a specific point mutant of Cytochrome C, thereby selectively eliminating its activation of Apaf-1 and other apoptotic components, while preserving its activity in the electron transport chain (12). By embryonic day (E) 14.5, these mice had severe overgrowth in multiple regions of the brain and insufficient skull development in comparison to WT counterparts, with these malformations resulting in embryonic or perinatal death for a majority of the mutant pups. Satellite glial cell (SGC) precursors have subsequently been characterized as a primary phagocyte population that clears excess apoptotic neurons, specifically in the embryonic dorsal root ganglion (DRG) (13). Isolated SGC precursors from E12.5 were found to express *Megf10* and *Pear1*, two homologs of the CED-1 phagocytic receptor in *Caenorhabditis elegans*. shRNA-mediated knockdown of *Megf10* and *Pear1* expression in cultured SGC precursors significantly impeded their capacity to engulf dying DRG neurons *in vitro* (13). These findings indicate that MEGF10 and PEAR-1 may be required for proper clearance in the setting of the developing nervous system, though further *in vivo* studies would be necessary to confirm this model. In contrast, a distinct phagocytic receptor, MERTK, is not required in the embryo, although it is expressed in the brain at E14.5 (14). Genetic ablation of *Tyro3, Axl*, and *Mertk* (TAM TKO) in mice had no discernible effect on embryonic viability or development, with TAM TKO mice maintaining normal leukocyte development and numbers up until postnatal day 28 (15). Yet as mentioned above, these receptors are critical for efferocytosis in adult mice in a number of tissues, including the brain and thymus (16–18). A comprehensive analysis of the expression of and requirement for efferocytosis receptors in embryonic vs. adult phagocytes could further enhance our understanding of whether specific apoptotic cell sensors are dedicated to homeostatic clearance.

The TAM family of receptor tyrosine kinases, consisting of TYRO3, AXL, and MERTK, represent one group of apoptotic cell sensors that recognize the common “eat-me” signal of PtdSer, but are differentially employed for engulfment by diverse phagocyte populations (16). Systematic evaluation of the *in vitro* phagocytic capacity of resident and thioglycollate-induced peritoneal macrophages deficient in either *Mertk, Axl*, or *Tyro3* underscored that all three TAM receptors can contribute to engulfment of apoptotic cells by peritoneal macrophages, although to differing extents; macrophages lacking *Mertk* had the most significant impairment in phagocytosis (19). Interestingly, bone marrow-derived dendritic cells (DCs) demonstrated a distinct reliance on *Axl* and *Tyro3* for engulfment, rather than *Mertk* (19). Such divisions are also reflected *in vivo*—*Axl* and *Tyro3* were not required for homeostatic removal of apoptotic cells in the thymus or the photoreceptor outer segments in the retina, while *Mertk* was essential for proper clearance (19). Recent studies have demonstrated that the receptor expression profile of phagocyte subsets is also dynamic in response to specific stimuli. Zagorska *et al*. characterized the differential regulation and utilization of AXL and MERTK phagocytic receptors by macrophages in either inflammatory or tolerogenic contexts (20). Treatment of bone marrow-derived macrophages (BMDMs) with Dexamethasone, an anti-inflammatory corticosteroid, elevated expression of MERTK and another PtdSer sensor *Bai1*, but not AXL. In contrast, a range of inflammatory signaling molecules, including lipopolysaccharide (LPS), polyinosinic:polycytidylic acid, IFNα, and IFNγ, were sufficient to enhance the expression of AXL in BMDMs, which expressed AXL only at low levels under normal culture conditions. In line with this, BMDMs lacking *Mertk* displayed severe defects in their baseline capacity to engulf apoptotic thymocytes, whereas *Axl*^−/−^ BMDMs only exhibited diminished uptake compared to WT BMDMs in inflammatory settings (20). This paradigm is also conserved in human phagocytes, as monocytes isolated from peripheral blood mononuclear cells upregulated MERTK expression in response to Dexamethasone or the combination of IL-10 and macrophage colony-stimulating factor (21). Such a “division of labor” is also not restricted to phagocytic receptor tyrosine kinases, as IL-4 was also shown to boost the expression of CD300f in a dose-dependent manner in macrophage subsets that typically do not express this receptor, including BMDMs cultured with IL-4 and peritoneal macrophages following *in vivo* administration of complexed IL-4 (22). CD300f levels in BMDMs were not sensitive to other cytokines tested, including the type II cytokine IL-13, which shares a receptor and has overlapping functions with IL-4 (23). Consequently, the sensitivity of apoptotic cell sensors to regulation by specific cytokines suggests that individual receptors may be equipped to help phagocytes respond to the presence of apoptotic cells in a given setting.

Intriguingly, additional signals in the tissue during infection may feedback to promote phagocytosis *via* specific phagocytic receptors, but without directly modulating receptor expression. Erdman *et al*. observed that human and murine macrophages displayed an increased capacity to clear either *Plasmodium falciparum-*infected or α-CD36-coated erythrocytes when pretreated with agonists to various Toll-like receptors (TLRs), including TLR2, TLR3, TLR4, and TLR9 (24). Uptake was dependent on CD36, a member of the class B scavenger receptor family; however, TLR stimulation did not alter surface expression of CD36 in the short timeframe of the assay (24). Thus, these results provide evidence for a level of cooperation between CD36 and various TLRs in order to potentiate engulfment. A similar synergy has also been described for uptake through integrins in the β_2_ subfamily, which are important for the phagocytosis of complement-opsonized particles. Again, stimulation with TNFα, LPS, or platelet activating factor did not have an effect on α_M_β_2_ expression by J774 murine macrophages (25). Instead, exposure to each of these inflammatory stimuli mediated activation of RAP1, a Ras-like GTPase that enhanced α_M_β_2_ binding to C3bi-coated erythrocytes; expression of a dominant-negative form of RAP1 in macrophage cell lines abolished their capacity to phagocytose these target cells (25).

### Signaling an Appropriate Response

While the downstream signaling pathways for phagocytic receptors are not yet fully defined, differences in the molecular players may enable distinct functional outputs by the phagocyte. BAI-1, TIM-4, and the TAM family of phagocytic receptors each recognize the same signal on apoptotic cargo (PtdSer), but partly diverge in the ensuing intracellular cascades that are activated. Immunoprecipitation assays have demonstrated that BAI-1, a G protein-coupled receptor, physically forms a complex with ELMO-1 and Dock-180. Altogether, these molecules function as a guanine nucleotide exchange factor for Rac, thereby driving phagosome formation. All components of this signaling complex were required for maximal engulfment, as silencing of endogenous *Elmo1* impaired the uptake of apoptotic thymocytes in *Bai1*-GFP transfected J774 murine macrophages (26). Other phagocytic receptors, including α_v_β_5_ and MERTK, have also been shown to engage the ELMO-1/Dock-180 machinery (27, 28), with constitutively active MERTK only phosphorylating p130^CAS^, an adaptor protein in this pathway, if α_v_β_5_ is coexpressed.

As discussed in further detail in Section “Specification Through Integration,” activated TAM receptors also have the capacity to trigger additional signaling cascades, like the phosphorylation of PLCγ2 (29), or the induction of *Socs1/3* to foster an active state of immunosuppression (30). In stark contrast, TIM-4 is thought to function solely as a tethering receptor, binding PtdSer *via* a metal-ion-dependent ligand binding site within its IgV domain (31) and securing apoptotic cargo on the surface of the phagocyte. In support of this, transient expression of either full-length *Tim4* or various versions of *Tim4* lacking the cytoplasmic tail all rendered LR73 fibroblasts more capable of engulfing apoptotic thymocytes than control fibroblasts (32). Moreover, ablation of *Tim4* prevented cultured peritoneal macrophages from binding FAS ligand-treated thymocytes, and transformation of the mouse B-cell line Ba/F3 with *Tim4* alone rescued the binding step of phagocytosis, but not internalization (33). Thus, TIM-4 requires pairing with other phagocytic receptors, such as MERTK, in order to mediate apoptotic cell engulfment in different phagocyte populations (33, 34). This cooperation adds a further layer of complexity to the apoptotic cell:phagocyte interface, as the activation of additional permutations of tethering and engulfing receptors could potentially generate distinctive combinations of intracellular signaling modules.

### Phagocyte Identity and Localization

Differences in the identity of the engulfing phagocyte may also account for variation in the resulting response to apoptotic cell recognition. The clearance of dying cells is primarily carried out by professional phagocytes—tissue-resident macrophages, monocyte-derived macrophages, and DCs—that are responsible for taking up cellular debris, sensing for any molecular patterns associated with pathogens, and processing and presenting antigen to activate the adaptive immune response. Despite the maintenance of resident professional phagocyte populations within the tissues, dedicated phagocytes and non-professional phagocytes, including neighboring epithelial cells, endothelial cells, and fibroblasts, are also indispensible for clearing apoptotic cells (35, 36). Cummings *et al*. ascertained that within the small intestinal lamina propria alone, three subsets of resident professional phagocytes contribute to the basal clearance of apoptotic intestinal epithelial cells (IECs) that are not shed off into the intestinal lumen (37, 38). Using a mouse model in which the Villin promoter drives expression of the diphtheria toxin (DT) receptor and GFP, the authors administered low levels of DT to induce cell death in IECs without significant inflammation, and subsequently tracked dying IEC uptake. While CD11b^+^ macrophages, CD11b^+^CD103^+^ macrophages, and CD103^+^ DCs each adopted a broadly immunosuppressive transcriptional signature in response to IEC engulfment, microarray data revealed distinctions in the precise genetic program; only two genes were consistently up- or downregulated amongst all three groups of phagocytes (37). Comparable resident mononuclear phagocyte subpopulations have been classified in other tissues, such as the kidney, which at steady-state already differ in their phagocytic capacity and expression patterns of cytokines, growth factors, and chemokine receptors (39). Collectively, these findings indicate that subsets of phagocytes, despite being exposed to the same microenvironment, maintain an intrinsic capacity to respond differently to apoptotic corpse engulfment.

## Specification through Integration

Phagocytes rarely are exposed to a single stimulus at once nor, as considered, do they react equivalently to a given signal under all circumstances. The integration of multiple, contemporaneous signals therefore represents a general mechanism through which cells could generate distinct, tailored outputs in response to their microenvironment. This integration could occur at the molecular and/or cellular level, and then serve to further regulate the immune response, such as through amplification or inhibition of specific effectors.

**Figure 1:**
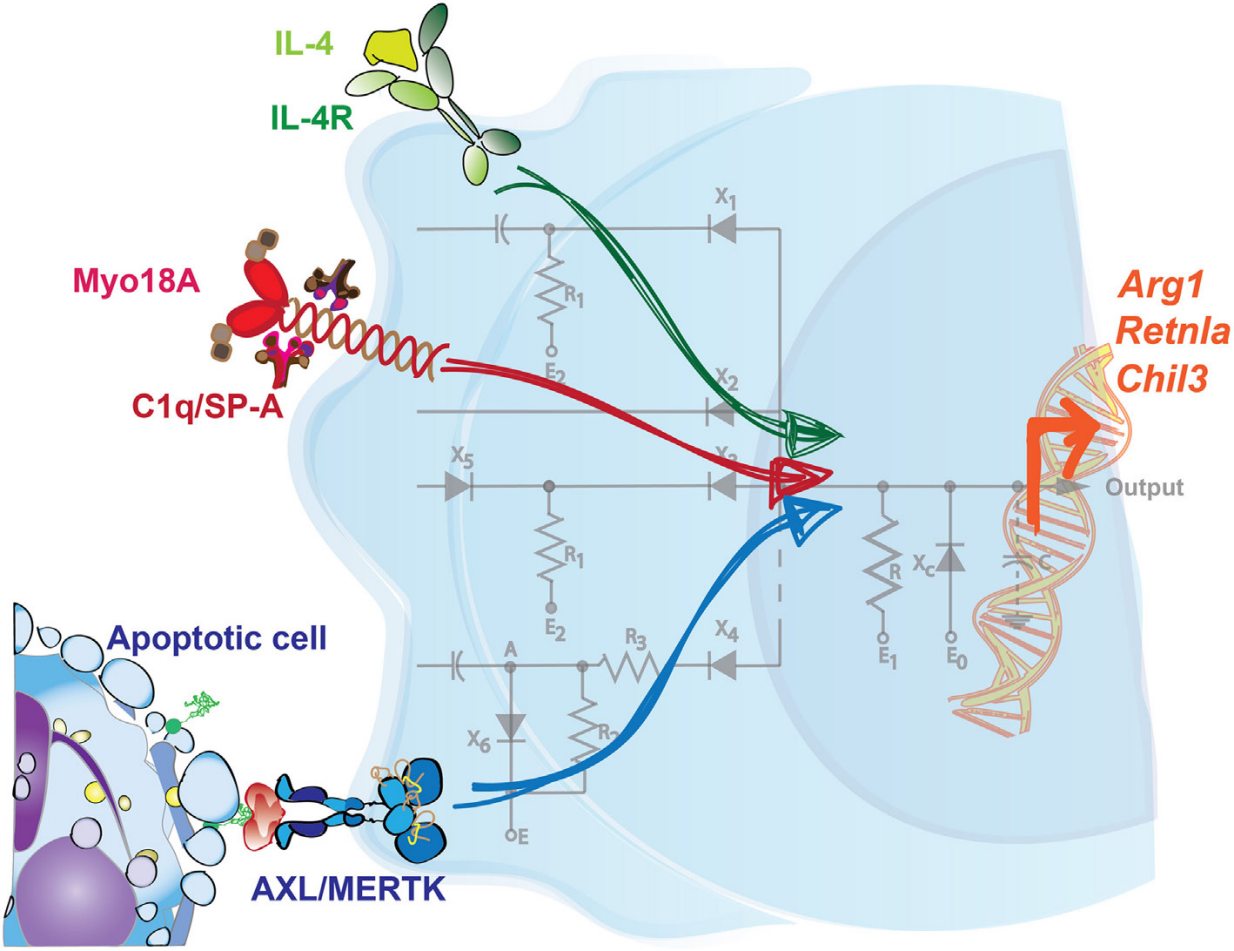
Integration of signals is required to drive a specific tissue repair program in macrophages. Sensing of IL-4 in the presence of apoptotic cells promotes the expression of key tissue repair factors in macrophages. Neither signal on its own is sufficient to induce this genetic program, which includes the upregulation of *Arg1, Retnla*, and *Chil3*. Additionally, C1q and surfactant protein-A (SP-A) are each sensed through Myo18A in distinct macrophage populations, and in conjunction with IL-4, prompts a similar set of tissue repair genes. Mirroring the configuration of a circuit, multiple inputs are therefore necessary to coordinate a tailored output or response by phagocytes.

One example of discrete signals combining to generate a specific output by phagocytic macrophages is the synergy between the sensing of apoptotic cells and of type 2 cytokines. Upon infection or injury, there is a significant increase in the magnitude of apoptotic cell death, with resident cells damaged by the insult dying off and infiltrating immune cells turning over. Multiple soluble ligands, like cytokines, are also produced at different phases of the inflammatory response, and phagocytes are also capable of sensing these factors. Work from our laboratory established that BMDMs, as well as macrophages in the lung, the intestine, the visceral adipose tissue, and the peritoneum, launch an efficient tissue repair response only when the sensing of IL-4/IL-13 occurs in the presence of apoptotic cells (Figure 1) (40). Importantly, this was not merely an additive effect of two parallel pathways—the presence of apoptotic cells alone did not induce the expression of some of the remodeling-associated genes analyzed. Integration of these two signals was shown to be dependent on the detectors AXL and MERTK, two phagocytic receptors expressed at both steady state and/or upon damage by resident and monocyte-derived macrophages. Genetic ablation of *Axl* and *Mertk*, or the administration of Annexin-V, which coats and obscures exposed PtdSer on apoptotic cells, were each sufficient to curtail this specialized tissue repair genetic program in macrophages in response to IL-4/IL-13. These findings demonstrate that apoptotic cell sensing can influence phagocyte activity in ways beyond its described immunosuppressive effect.

Interestingly, while the maximal induction of remodeling-associated genes like *Arg1, Retnla*, and *Chil3* were reliant on macrophages receiving these two signals, other IL-4-mediated responses were not. Gene set enrichment analysis of BMDMs treated with IL-4 alone identified categories such as pattern recognition signaling pathways, regulation of cytokine production, chemotaxis, and the defense response, all of which were unaffected by an additional treatment of the BMDMs with exogenous apoptotic cells. Like other cytokines, IL-4 and IL-13 are produced at multiple phases of the immune response and have pleiotropic effects, including driving cell proliferation, antigen presentation, antimicrobial activity, and this induction of tissue remodeling (41–43). Collectively, these results shed light on a role for apoptotic cells in specifying the action of IL-4 on one of its target cell populations. In this way, the requirement of both signals functions as a checkpoint, restricts the expression of repair factors to both an appropriate time and location, and helps to avoid the potentially detrimental effects of aberrant remodeling. While the effects of this signal integration have been characterized, further investigation is needed to determine the precise molecular players that interact downstream in the IL-4/IL-13 and TAM signaling pathways.

It is important to note that additional tissue-specific factors have also been described to promote the resolution program in phagocytes in response to IL-4. Minutti *et al*. recently identified surfactant protein-A (SP-A) and complement component C1q as secondary stimuli that enhance the proliferative and tissue-remodeling capacity of IL-4-activated macrophages from the lung and the peritoneal cavity/liver, respectively (Figure 1) (44). In support of this, SP-A or C1q were each necessary for extensive repair responses in settings of acute and chronic damage, like with *Listeria monocytogenes* infection or in the Dineal PD-4 model of peritoneal fibrosis. *Sp-a*^−/−^ mice, for example, exhibited severe immunopathology in the lung and increased worm burden in the intestine following infection with the helminth *Nippostrongylus brasiliensis* in comparison to their WT counterparts. Integration of these pathways is at least partially mediated through myosin 18 A (Myo18A); *in vivo* delivery of IL-4 was shown to increase the expression of this shared receptor for SP-A and C1q in macrophages isolated from the lung, liver, and peritoneal cavity (44).

As referenced before, uptake of apoptotic cells by phagocytes has been shown to suppress the response to inflammatory stimuli, like TLR ligands (45–51). Engulfment of apoptotic cells by immature murine bone marrow-derived DCs was shown to dampen the response to LPS, with specific reduction of IL-12 secretion and CD86 expression, but not of other proinflammatory cytokines or costimulatory molecules, like TNFα and CD40 (50). In a similar manner, ingestion of apoptotic corpses by LPS-stimulated human or murine macrophages diminished secretion of proinflammatory molecules TNFα and IL-1β, but also enhanced release of anti-inflammatory signals, such as TGF-β1 and PGE-2 (45, 48, 49, 51). Thus, beyond suppressing proinflammatory events, integration of TLR signaling and apoptotic cell sensing actively promotes the generation of an anti-inflammatory environment.

The precise mechanism of integration of TLR signaling and uptake of apoptotic cells still needs to be fully elucidated. Intracellular lipid sensors have been described to contribute to this immunosuppressive effect of apoptotic cells (52, 53). Additionally, integration of phagocytic receptor and TLR signaling with cytokine signaling has been characterized as another strategy for dampening the inflammatory response by phagocytes. The receptor tyrosine kinase AXL, which is highly expressed by DCs (20, 30), was found to physically associate with the R1 chain of the type I IFN receptor (IFNAR1) upon administration of its ligand Gas6 (30). Engagement of and interaction between these two receptors led to the activation of STAT1, which in turn triggered SOCS1 and SOCS3 to limit both cytokine and TLR signaling (30). This cooperation highlights how the pairing of apoptotic cell sensors with other detectors not only can promote a specialized transcriptional program, but also can negatively regulate phagocyte activation in a specific manner. The extent to which phagocytic receptor signaling integrates with other cytokine pathways, and whether these are broader features of all phagocytic receptors, remain to be fully explored.

## Concluding Thoughts

**Figure 2:**
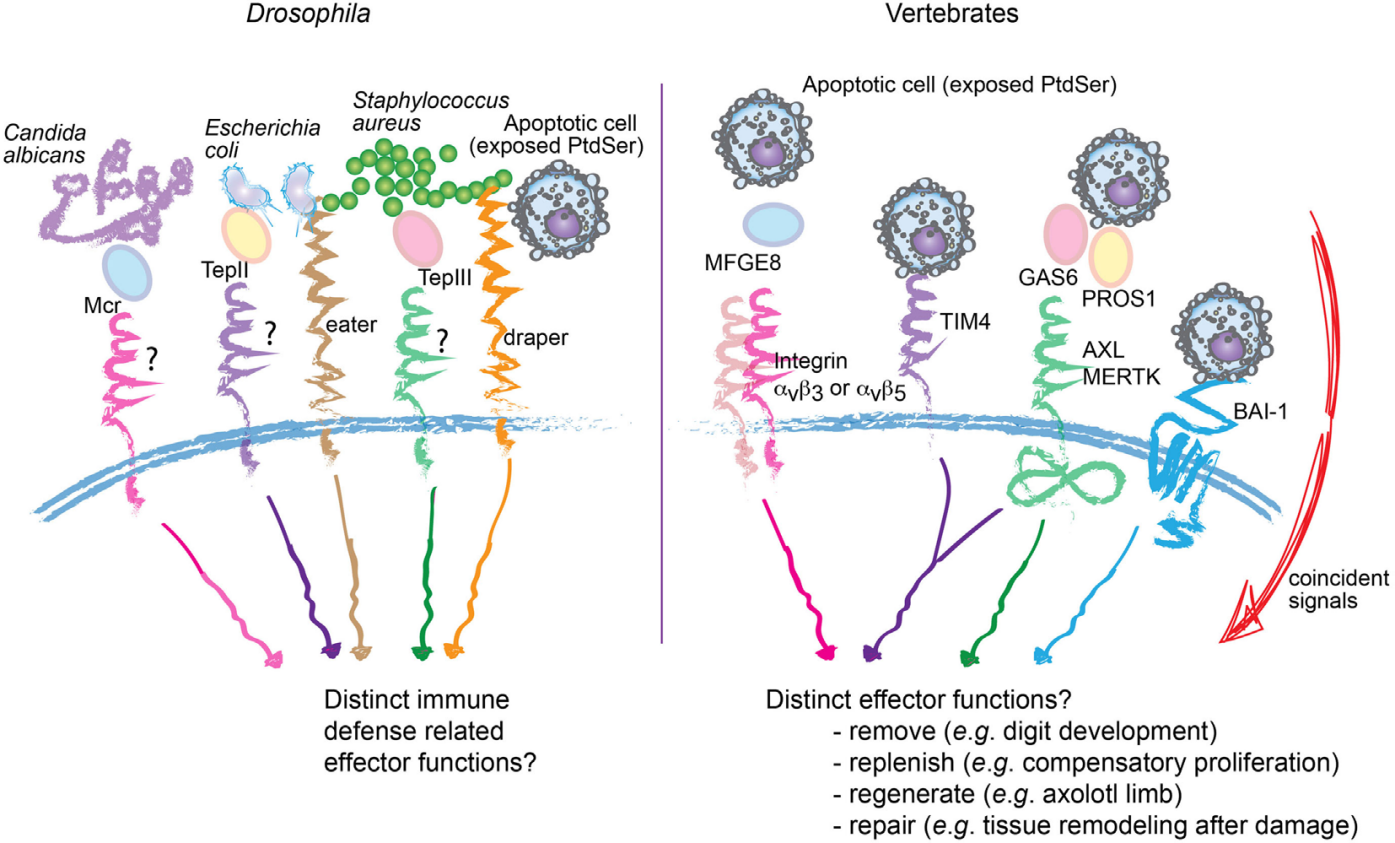
Proposed specificity, rather than redundancy, in apoptotic cell recognition and engulfment. Comparative representation of molecular mediators involved in the recognition and engulfment of bacteria or apoptotic cells by phagocytes, in *Drosophila* (left) and vertebrates (right). In *Drosophila*, specific bacteria can be opsonized through secreted Mcr, Tep II, or Tep III before engulfment by hemocytes. Similarly, multiple phagocytic receptors in vertebrates, including integrins α_v_β_3_, α_v_β_5_ and the receptor tyrosine kinases AXL and MERTK (right) also require “opsonization” of the apoptotic cargo. This raises the possibility of selective disposal and tailored effector functions. Consistent with this proposition, in vertebrates, not only are the number of phagocytic receptors and ligands expanded but also their engagement is integrated with specific signals from the microenvironment.

The functional diversification of apoptotic cell removal in mammals is consistent with the evolutionary expansion of PtdSer receptors. While efferocytosis itself is conserved from *C. elegans* to mammals, at least some of the mammalian receptors such as BAI-1, TIM4, and the TAMs do not have orthologs in *C. elegans*. The argument that increased redundancy of these receptors simply ensures efficient removal of dead cells, as the risk for autoimmunity increases with evolution, cannot be formally ruled out. Intriguingly, TAM, integrin, and MEGF10-mediated efferocytosis require “opsonization” of the apoptotic cargo with their respective ligands, including PROS1/GAS6, MFGE8, or C1q (54) (Figure 2). This system seems analogous to that seen in *Drosophila* hemocytes, in which secreted Mcr, Tep II, or Tep III opsonize *Candida albicans, Escherichia coli*, or *Staphylococcus aureus*, respectively, during phagocytosis (55). The selective advantage of such a system is difficult to understand simply on the basis of redundancy; rather, it favors a paradigm wherein layers of specificities can be built in. Overall, evolutionary expansion of phagocytic receptors may have enabled cargo-selective disposal and the resulting specification in phagocyte response. Further inquiry of this topic may permit the establishment of a “code” (56), through which knowledge of the various input signals from the dying cell and the surrounding microenvironment can predict the functional output of the phagocyte.

## Author Contributions

All authors listed have made a substantial, direct, and intellectual contribution to the work and approved it for publication.

## Conflict of Interest Statement

The authors declare that the research was conducted in the absence of any commercial or financial relationships that could be construed as a potential conflict of interest.
